# Post-Transplant Lymphoproliferative Disorders: Role of Viral Infection, Genetic Lesions and Antigen Stimulation in the Pathogenesis of the Disease

**DOI:** 10.4084/MJHID.2009.018

**Published:** 2009-12-14

**Authors:** Daniela Capello, Gianluca Gaidano

**Affiliations:** Division of Hematology, Department of Clinical and Experimental Medicine and BRMA, Amedeo Avogadro University of Eastern Piedmont, Novara, Italy

## Abstract

Post-transplant lymphoproliferative disorders (PTLD) are a life-threatening complication of solid organ transplantation or, more rarely, hematopoietic stem cell transplantation. The majority of PTLD is of B-cell origin and associated with Epstein–Barr virus (EBV) infection. PTLD generally display involvement of extranodal sites, aggressive histology and aggressive clinical behavior. The molecular pathogenesis of PTLD involves infection by oncogenic viruses, namely EBV, as well as genetic or epigenetic alterations of several cellular genes. At variance with lymphoma arising in immunocompetent hosts, whose genome is relatively stable, a fraction of PTLD are characterized by microsatellite instability as a consequence of defects in the DNA mismatch repair mechanism. Apart from microsatellite instability, molecular alterations of cellular genes recognized in PTLD include alterations of cMYC, BCL6, TP53, DNA hypermethylation, and aberrant somatic hypermutation of protooncogenes. The occurrence of IGV mutations in the overwhelming majority of PTLD documents that malignant transformation targets germinal centre (GC) B-cells and their descendants both in EBV–positive and EBV–negative cases. Analysis of phenotypic markers of B-cell histogenesis, namely BCL6, MUM1 and CD138, allows further distinction of PTLD histogenetic categories. PTLD expressing the BCL6+/MUM1+/-/CD138− profile reflect B-cells actively experiencing the GC reaction, and comprise diffuse large B-cell lymphoma (DLBCL) centroblastic and Burkitt lymphoma. PTLD expressing the BCL6−/MUM1+/CD138− phenotype putatively derive from B-cells that have concluded the GC reaction, and comprise the majority of polymorphic PTLD and a fraction of DLBCL immunoblastic. A third group of PTLD is reminiscent of post-GC and preterminally differentiated B-cells that show the BCL6−/MUM1+/CD138+ phenotype, and are morphologically represented by either polymorphic PTLD or DLBCL immunoblastic.

## Introduction:

**Figure 1.**
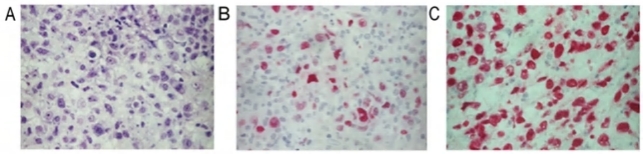
***Morphology and phenotype of PTLD***. **(A)** P-PTLD consisting mainly of small- and medium-sized lymphoid cells (Giemsa staining). **(B)** PTLD with diffuse large B cell morphology displaying the BCL6^+^/MUM1^−^/CD138^−^ phenotypic pattern. Tumour cells show nuclear staining pattern with the anti BCL6 MoAb. **(C)** P-PTLD displaying the BCL-6^−^/MUM1^+^/CD138^−^ phenotypic pattern. Most neoplastic cells show strong nuclear immunoreactivity with the anti MUM1 antibody. (Paraffin-embedded tissue sections, magnification x 400).

Post-transplant lympho-proliferative disorder (PTLD) is one of the most serious complications of immunosuppression in patients undergoing both solid organ and hematopoietic stem cell (HSC) transplantation, contributing significantly to morbidity and mortality in this group of patients^1^–^4^. PTLD encompass a heterogeneous group of lymphoproliferative diseases, ranging from reactive, polyclonal hyperplasia, to highly aggressive monomorphic proliferations which may be indistinguishable from aggressive lymphomas^5^,^6^. According to the WHO classification^7^, PTLD may be classified into: (i) early lesions, generally represented by EBV driven polyclonal lymphoproliferations, and (ii) true monoclonal diseases, including polymorphic PTLD (P-PTLD) and monomorphic PTLD; the latter further distinguished into Burkitt lymphoma (BL), diffuse large B-cell lymphoma (DLBCL) and Hodgkin lymphoma (Figure 1).

There is a known relationship between Epstein Barr Virus (EBV) and PTLD, given that the EBV genome is found in approximately 80% of PTLD specimens^8^,^9^. In these cases, the pathogenesis of PTLD is associated with the uncontrolled proliferation of EBV infected B-cells in the absence of EBV-specific cellular immune response^9^. PTLD are, however, not exclusively associated with EBV infection, as EBV-negative PTLD, with a preference to develop late after transplantation, are frequently reported^10^–^12^.

The risk of developing PTLD varies greatly, depending upon the type of transplanted organ, the patient’s age at transplantation, and the immune-suppressive regimen used. In HSC transplant recipients, the incidence of PTLD is 0.5% after HLA-matched noncomplicated transplants and 25% after T-cell-depleted highly immunesuppressed transplants^13^. In the case of solid organ transplantation, the overall incidence of PTLD is 1–5%^14^–^16^. The disease arises in 1–5% kidney and liver transplant recipients, 5–15% heart and heart-lung transplant patients, and 10–15% intestinal transplant recipients^14^–^16^. PTLD occur more commonly in pediatric patients than in adults^17^. The higher incidence in children is thought to result from the fact that they have a greater likelihood of being EBV-naïve recipients of EBV-seropositive graft^17^. PTLD is observed more frequently in the first year following transplantation, when the recipient is more severely immunocompromised. However, as the prognosis improves for individuals receiving solid organ transplant, a long-term risk of PTLD development late after transplantation is increasingly recognized^11^,^12^,^14^–^16^.

PTLD share several features with other immunodeficiency-related lymphomas^5^–^7^. These common features include a preferential representation of non-Hodgkin lymphoma (NHL) versus Hodgkin lymphoma, B-cell lineage derivation, involvement of extranodal and unusual sites, aggressive histopathology, aggressive clinical behavior, and frequent association with EBV infection. Despite these common features, PTLD display a high degree of histogenetic and molecular heterogeneity^18^–^21^. Early-onset PTLD, occurring within 1 year after transplantation, are mainly polyclonal or monoclonal polymorphic B-cell proliferations, frequently associated with Epstein–Barr virus (EBV) infection. Conversely, most late-onset PTLDs are monoclonal lymphoid malignancies carrying EBV infection only in a fraction of cases^1^–^4^,^11^,^12^.

Although it is generally assumed that most PTLD occurring after solid organ transplantation arise from lymphoid cells of the recipient (R-PTLD), an increasing number of case reports suggest that, particularly in liver transplant recipients, a considerable fraction of PTLD arise from donor B-cells (D-PTLD)^22^. In liver transplant patients, D-PTLD and R-PTLD differ significantly for timing and clinical presentation. Generally, D-PTLD are early-onset, EBV-driven lymphoproliferations that, at diagnosis, are clinically and histologically confined to the hepatic hilum. On the contrary, R-PTLD are mainly late-onset lymphoproliferations that, at diagnosis, are widespread diseases with involvement of multiple nodal and extranodal sites^22^.

This review will focus on the molecular pathogenesis and histogenesis of PTLD occurring in patients undergoing solid organ transplantation, with special emphasis on the role of viral infection, cellular molecular lesions and antigen stimulation in the pathogenesis of the disease.

## Molecular Pathogenesis of Ptld

### Viral infection:

Oncogenic viruses known to be involved in PTLD pathogenesis include EBV and human herpesvirus type-8 (HHV-8). Both EBV and HHV-8 act predominantly through direct mechanisms, i.e. the virus is able to directly infect the tumor clone and exerts a transforming effect upon B-cells. Viral infection in PTLD exploits several strategies to ensure persistent infection, namely prevention of death of infected cells, enhancement of their proliferation to maintain the infected reservoir, and evasion of the immune system^9^,^23^–^25^.

### EBV:

Several lines of evidence suggest that EBV infection has a major pathogenetic role in PTLD. First, EBV infects 60–80% PTLD, including 100% early PTLD, and 80–100% post-transplant HL^24^,^25^. Second, in many cases of monomorphic PTLD, EBV infection is monoclonal, consistent with the hypothesis that the virus has been present in the tumor progenitor cells since the early phases of clonal expansion^18^. Third, EBV infected B-cells are present in increased number in blood and tissues of patients who subsequently develop PTLD^26^,^27^. Furthermore, decrease of EBV-specific cytotoxic T-cells and increase in EBV viral load is strongly associated with PTLD development^28^,^29^. Fourth, treatment of PTLD with autologous EBV-specific cytotoxic T-cells may result in viral load control and tumor size reduction^30^. Finally, several viral genes expressed during latent infection of PTLD have transforming activity for B-cells^9^,^31^–^33^.

EBV is a double strand DNA virus belonging to the γ-herpesvirus family that benignly infects over 95% of the human population for life^31^–^33^. EBV targets B lymphocytes and, after acute infection, the virus DNA forms a circle and persists as an episome in the nuclei of resting memory B-cells establishing a latent infection^31^–^33^. In latently infected B-cells, EBV encodes a series of viral proteins that interact with or exhibit homology to a variety of signal transducers, cytokines and antiapoptotic human molecules. These proteins are EBV nuclear antigens (EBNA)1, EBNA2, EBNA3A, EBNA3B, EBNA3C, EBNA-LP and the latent membrane protein (LMP)1, LMP2A and LMP2B. Beside these proteins, EBV-encoded non-translated RNAs (EBER) are transcribed in latently infected B-cells^31^–^33^. Based on the pattern of expression of the latency genes, three types of latent infection have been described: i) latency I, that is defined by the expression of EBER and EBNA1; ii) latency II, that is characterized by the expression of EBER, EBNA1, LMP1, LMP2; and iii) latency III, the “growth program”, denoted by the expression of EBER, all EBNAs, LMP1 and LMP2^31^–^33^. In PTLD, all three latency programs may be observed.

EBNA1 is a DNA-binding nuclear phosphorprotein, that is required for replication and maintenance of the episomal EBV genome^31^–^33^. EBNA1 is also a cis-acting inhibitor of MHC class I-restricted presentation and an inhibitor of antigen processing via the ubiquitin/proteosome pathway^34^. Directing EBNA1 expression to B cells in transgenic mice has been shown to result in B-cell lymphomas, suggesting that EBNA1 might have a direct role in lymphomagenesis cooperating with MYC^35^.

EBNA2 is a transcriptional coactivator that regulates both viral latency genes, for example LMP1 and LMP2, and many cellular genes involved in proliferation and survival, including cMYC^31^–^33^,^36^. EBNA2 does not bind directly to the DNA but interacts with other transcription factors, namely the viral transcription factor Cp1 and the cell transcription factor RBP-Jk involved in the NOTCH1 signaling pathway^37^. This pathway is well known to be involved in lymphomagenesis, since NOTCH1 is a proto-oncogene frequently activated by mutation/translocation in T-cell lymphoblastic lymphoma^38^.

Two main types (1 and 2) of EBV are distinguished on the basis of sequence variation in the EBNA proteins. Although type 2 EBV frequently infects immunosuppressed individuals, no clear correlation has been identified between EBV strain and lymphoma development^39^–^44^.

**Figure 2.**
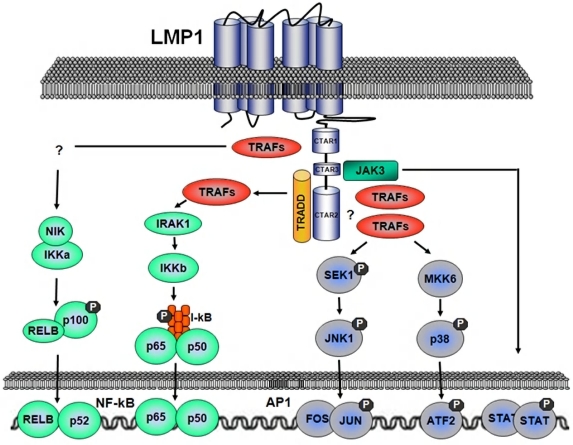
***The role of LMP1 in promoting survival in PTLD***. LMP1 is a transmembrane protein consisting of a short N-terminal cytoplasmic domain, six transmembrane-spanning domains and a C-terminal cytoplasmic domain. LMP1 shares no significant overall homologies with any known cellular protein. The transmembrane domains of LMP1 have the intrinsic feature to oligomerize spontaneously within the plasma membrane. This autoaggregation of LMP1 molecules mimics the crosslinking of receptors of the TNF-R family induced by a ligand (e.g. TNF-R1 by TNF□□□CD40 by CD40L). As a result, LMP1 is constitutively active independent of the binding of a ligand. Three subdomains in the cytoplasmic C-terminus of LMP1 (CTAR 1, 2, 3) serve as platforms for the binding of signalling molecules and for induction of signal transduction cascades. Both CTAR1 and CTAR2 participate in the induction of the anti-apoptotic transcription factor NF-kB. CTAR1 provides a docking site for TNF-R-associated factors (TRAFs). CTAR1 signalling induces the non-canonical NF-kB pathway via the NF-κB-inducing kinase (NIK) and the Inhibitory-kB Kinase (IKK) □. CTAR2 does not bind TRAFs directly but triggers the classical NF-kB pathway through direct interaction with the TNF-R-associated death domain protein (TRADD). TRADD, in the contest of TNF-R1 complex, normally signals cell death, whereas, in the context of LMP1, signals cell growth. Also the mitogenic c-Jun N-terminal kinase 1 (JNK1) / activator protein 1 (AP1) pathway is activated by LMP1. AP1 is a dimer of JUN/JUN or JUN/FOS proto-oncoproteins. AP1 is solely triggered at CTAR2. The JAK3/STAT signalling cascade, known to be involved in the control of cell proliferation, is triggered by the CTAR3 domain which is located between CTAR1 and CTAR2. It has been demonstrated that the p38 MAPK pathway is also induced by LMP1. p38 MAPK mediates cytokine induction by LMP1.

LMP1 is the major transforming protein of EBV (Figure 2)^31^–^33^. LMP1 is an integral membrane protein expressed on the surface of infected B-cells and involved in transformation by acting as a constitutively active substitute for CD40, a receptor that physiologically provides a signal for proliferation and survival to B-cells^45^. LMP1 mimics CD40 by binding the same cytoplasmic signal transduction molecules, namely the tumor necrosis factor receptor associated factors (TRAFs)^46^. TRAFs, in turns, activate at least four signaling pathways represented by NF-kB, c-Jun N-terminal kinase 1 (JNK1) / activator protein 1 (AP1), p38 mitogen-activated protein kinase and the JAK3/STAT pathway. These molecules affect diverse signaling cascades that lead to enhanced expression of B-cell adhesion molecules, activation markers, cMYC and the antiapoptotic factors BCL2 and A20^47^–^50^.

LMP1 appears to play a critical role in the pathogenesis of PTLD derived from donor B-cells. In a recent study from our group^22^, all D-PTLD associated with EBV infection expressed the viral oncoproteins EBNA2 and LMP1. Notably, seven out of nine D-PTLD were infected by EBV variants with deletion of the carboxy terminus of LMP1. In particular, five cases showed the Δ69-LMP1 variant, which was rarely detected in a consecutive series of PTLD from the same geographical area and infrequently found in the Italian population. LMP1-deleted variants have been reported to be associated with HIV-related Hodgkin’s lymphoma, whereas association with PTLD is currently controversial^39^–^44^. The high prevalence of LMP1-deleted variants in D-PTLD could be related to the higher transforming activity displayed in vitro by these EBV variants^47^. Nevertheless, it remains to be determined whether currently unidentified recipient-related factors may have a role in favoring the generation of D-PTLD infected by LMP1-deleted variants, like the inability of the recipient immune system to recognize and destroy B-cells expressing LMP1 variants that potentially lack some epitopes.

**Figure 3.**
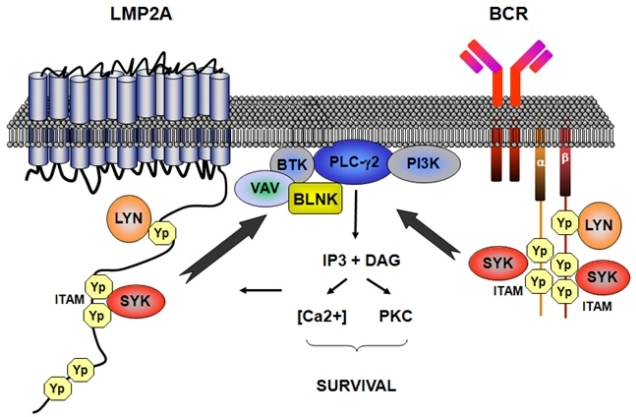
***Signalling relationship between LMP2A and the BCR***. LMP2A consists of cytoplasmic amino-terminal and carboxy-terminal domains linked by 12 transmembrane sequences with no significant extracellular domain. The amino-terminal domain of LMP2A contains the same immunoreceptor tyrosine-based activation motifs (ITAMs) found in the α- and β-chains of the B-cell receptor (BCR). Both LMP2A and the BCR associate with LYN, a member of the Src family of tyrosine kinases. Phosphorylation of tyrosine residues within the ITAM by LYN leads to recruitment of the SYK tyrosine kinase and the canonical downstream BCR signalling events. LMP2A signalling does not cause B cells to grow, but delivers the tonic signal that is essential for the survival of all B cells. BLNK, B-cell linker protein; DAG, diacylglycerol; [Ca2+], intracellular Ca2+; IP3, inositol-1,4,5-trisphosphate; PI3K, phosphatidylinositol 3-kinase; PLC-γ2; phospholipase C-γ2; PKC, protein kinase C; Yp, phoshorylated tyrosine.

Other EBV-encoded genes, though not strictly transforming, may also be involved in lymphomagenesis^31^–^33^. LMP2A is an integral membrane protein that contains an immunoreceptor tyrosine-based activation motif (ITAM) (Figure 3)^51^,^52^. This motif is similar to that present in B cell receptor (BCR) coreceptors CD79a and CD79b, and transmits activating signals after BCR stimulation. LMP2A binds and thus sequesters tyrosine kinases from the BCR, resulting in inhibition of BCR signaling^51^. This prevents unwanted antigen-triggered activation of infected B-cells, that would otherwise cause entry into lytic cicle. On the other hand, LMP2A is also able to stimulate the BCR-associated tyrosine kinases providing an important survival signal to B-cells^52^. EBER1 and 2 are short strand non-coding RNAs expressed in all forms of latent infection. EBERs seem to be involved in inducing autocrine IL-10 secretion by BL cells, that might stimulate growth of infected neoplastic B-cells and suppress cytotoxic T-cells^53^. In addition, EBERs might mediate resistance of BL cells to interferon-α (IFNα)^54^.

### Human Herpes Virus 8 (HHV-8):

HHV-8 is a double strand DNA virus belonging to the γ□-herpesvirus family. Similar to EBV, HHV-8 establishes a lifelong latent infection in which the viral DNA persists as an episome in the nuclei of infected cells^55^,^56^. HHV-8 infects 100% primary effusion lymphomas (PEL) arising in transplanted patients^7^,^57^. A number of HHV-8 genes are homologous to human genes involved in proliferation, anti-apoptosis, angiogenesis and cytokines that are able to transform human cells in vitro and/or in vivo, and are potentially involved in lymphomagenesis^55^,^56^. Post-transplant PEL are characterized by latent HHV-8 infection and express a restricted pattern of HHV-8 encoded genes^57^,^58^. HHV-8 encoded latency-associated nuclear antigen 1 (LANA1) interacts with p53 and suppresses the p53 transcriptional activity and ability to induce apoptosis^59^. Furthermore, LANA1 binds to the Rb protein, thus releasing the transcription factor E2F that upregulates genes involved in cell cycle progression^60^. Finally, LANA1 is able to induce expression of IL-6 through interaction with AP1 transcription factor^61^. The v-cyclin gene encodes a homologue of human cyclin D2^62^. Similar to cyclin D2, v-cyclin contributes to Rb phosphorrylation by activating cyclin-dependent kinases. Phosphorylated Rb then releases the E2F transcription factor, thus cooperating with LANA1 in inducing cell cycle acceleration^62^. In contrast to human cyclin D2, v-cyclin is resistant to inhibition by p16, p21 and p27, a family of cyclin dependent kinase inhibitors that physiologically block cell cycle progression^63^–^65^. Overall, v-cyclin permits to circumvent normal cell cycle checkpoints, and leads to constitutive cell cycling.

### Simian Virus 40 (SV40):

The involvement of simian virus 40 (SV40) in PTLD pathogenesis has been a matter of recent debate. Based on the paradigm of other lymphoma-related viruses, SV40 displays several features predicting a putative pathogenetic role. Although two initial reports described a high prevalence of SV40 in NHL, including immunodeficiency-related NHL, the association between SV40 and PTLD and, more in general, NHL has been subsequently denied by large molecular, immunohistochemical and serological studies^66^–^70^.

### Hepatitis C Virus (HCV):

Several epidemiological studies and meta-analysis have underlined the association between HCV infection and NHL in the immunocompetent host. The current pathogenetic hypothesis holds that HCV may act on B-cells indirectly through chronic antigen stimulation, as suggested by the identification of molecular clues of antigen stimulation in HCV-related NHL and by the expression of HCV specific IGV in a fraction of HCV-related NHL^71^. Although there are a number of case reports of PTLD occurring in patients positive for HCV, few studies have investigated the relationship between HCV and PTLD systematically, and the results are conflicting^72^–^74^.

## Molecular alteration of cellular genes:

Infection by oncogenic viruses is a pathogenetic mechanism often necessary, but not sufficient to develop monoclonal PTLD. Progression to lymphoma requires the accumulation of genetic or epigenetic alterations of cellular genes. At variance with lymphoma arising in immunocompetent hosts, whose genome is relatively stable, a fraction of PTLD are characterized by microsatellite instability as a consequence of defects in DNA mismatch repair mechanisms^75^. These cases are characterized by a mutator phenotype, thus accumulating mutations in several genes including the proapoptotic factors BAX and CASPASE 5, and the DNA repair gene RAD50. The explanation to why PTLD and other types of immunodeficiency-related lymphoma display a mutator phenotype, that is otherwise rarely observed in NHL of immunocompetent hosts, remains speculative. One hypothesis is that the mutator phenotype may generate numerous neoantigens at the tumor cell surface as a consequence of mutations affecting various genes. In the context of immunodeficiency, the host immune system might be less prone to recognize and eliminate such highly immunogenic lymphoma cells. Apart from microsatellite instability, molecular alterations of cellular genes recognized in PTLD include alterations of cMYC, BCL6, p53, DNA hypermethylation, and aberrant somatic hypermutation^76^.

### cMYC:

Analogous to BL arising in immunocompetent or HIV-infected hosts, chromosomal breaks at 8q24 are found in 100% post-transplant BL^5^,^7^. Chromosomal translocations cause cMYC deregulation by at least two distinct mechanisms^76^. First, translocated cMYC alleles are juxtaposed to heterologous regulatory elements derived from Ig loci^76^. Second, the regulatory regions of cMYC are consistently affected by structural alterations that are supposed to modify their responsiveness to cellular factors regulating cMYC expression^76^. Oncogenic conversion of cMYC also stems from amino acid substitutions in cMYC exon 2^76^. These mutations affect the amino-terminal transcriptional activation domain of the gene and allow escape from the p107-mediated modulation of the cMYC transactivator domain. The cMYC proto-oncogene promotes proliferation through different mechanisms, that include upregulation of genes involved in cell cycle control, downregulation of growth arrest genes, activation of telomerase reverse transcriptase (TERT), induction of protein kinase A and increase of lactate dehydrogenase-A gene, whose product participates in normal anaerobic glycolysis and is necessary for the growth of a cell mass with an hypoxic internal microenvironment^77^–^79^.

### BCL6:

BCL6 is a transcriptional repressor containing the POZ domain that is homologous to domains found in several other zinc-finger transcription factors^76^. BCL6 is needed for GC development and survival, whereas its downregulation may be necessary for further differentiation of B-cells. Rearrangements of BCL6 are found in 20–40% of DLBCL of immunocompetent hosts and HIV-DLBCL, but seldom occur in PTLD^80^. Conversely, in about 50% PTLD, the BCL6 gene is affected by multiple, often biallelic, mutations introduced by the SHM mechanism that selectively cluster within the noncoding regions of the gene^21^,^81^. The DNA sequences most frequently affected by mutations lie near the BCL6 promoter region and overlap with the major cluster of chromosomal breaks at 3q27, suggesting that mutations and rearrangements may be selected for their ability to alter the same region, which, conceivably, regulates the normal expression of BCL6^82^.

### TP53:

Mutations of TP53 are detected in a fraction of post-transplant DLBCL^18^. Missense mutations of TP53 usually result in the inability to transactivate its target genes, and downregulate expression of p21, a cyclin kinase inhibitor that neutralizes the activity of cyclin E^76^.

### Aberrant DNA methylation:

Aberrant hypermethylation of CpG islands is an epigenetic alteration that causes repression of gene transcription and represents a mechanism for tumor suppressor gene inactivation alternative to mutations/deletions^83^. Aberrant promoter hypermethylation has been documented as a relevant mechanism of lymphomagenesis in transplanted patients, targeting multiple and functionally heterogeneous lymphoma-related genes^84^. Hypermethylation of O6-methylguanine-DNA methyltransferase (MGMT) targets approximately 60% monomorphic PTLD^84^. MGMT is a DNA repair gene that removes mutagenic and cytotoxic adducts introduced in the DNA from environmental and therapeutic alkylating agents^85^. The potential role of MGMT in lymphoma stems from the fact that MGMT inactivation favors lymphomagenesis in knockout mice. Consistent with the protective function of MGMT against spontaneous and alkylator-induced G to A transitions in human DNA, MGMT inactivation may cause tumors by generating genetic instability and acquisition of p53 and RAS point mutations^85^. Hypermethylation of death-associated protein kinase (DAP-k) occurs in 75% monomorphic PTLD^84^. DAP-k is a pro-apoptotic serine-threonine kinase involved in the extrinsic pathway of apoptosis initiated by INFγ, TNFα and Fas ligand^86^. In addition, DAP-k also counteracts cMYC induced transformation by activating the p53 checkpoint and favoring cMYC induced apoptosis^86^. Consequently, inactivation of DAP-k prevents apoptosis triggered by death receptors and weakens the apoptotic response secondary to cMYC activation. The p73 gene is a candidate tumor suppressor gene sharing structural and functional similarity with p53 and involved in cell cycle control and apoptosis. Hypermethylation of p73 occurs in approximately 20% PTLD^84^.

### Aberrant somatic hypermutation:

Normally, the somatic hypermutation (SHM) process targets IGV genes of GC B-cells^87^. In over half of DLBCL, the SHM process appears to misfire and aberrantly target multiple proto-oncogenes implicated in the pathogenesis of lymphoid malignancies (PIM1, PAX5, RhoH/TTF and cMYC)^88^. PIM1 encodes a serine-threonine kinase and is occasionally involved in DLBCL associated chromosomal translocations^89^; PAX5 encodes a B-cell specific transcription factor essential for B-lineage commitment and differentiation and involved in translocations in about 50% of lymphoplasmacytic lymphoma^90^; RhoH/TTF encodes a small GTP-binding protein belonging to the RAS superfamily and is involved in rare instances of lymphoma translocations^91^. Mutations affecting PIM1, PAX5, RhoH/TTF and cMYC recapitulate the molecular features of physiological SHM, but they do not occur at a significant level in normal GC B-cells, suggesting a malfunction of SHM associated with DLBCL^88^. On these basis, this phenomenon has been termed aberrant SHM^88^. In PTLD, aberrant SHM is not restricted to DLBCL, and appears to be involved in the pathogenesis of lymphomas originating from both recipient and donor B-cells^22^,^92^. Based on the distribution and type of mutations, aberrant SHM may alter the function of PIM1, PAX5, RhoH/TTF and cMYC with two modalities^89^–^91^. First, because mutations cluster around the gene regulatory regions, mutations may deregulate gene transcription. Second, a subset of mutations of cMYC and PIM1 lead to aminoacid substitutions, and, consequently, may alter the biochemical and/or structural properties of the protein.

### Antigen stimulation:

**Figure 4.**
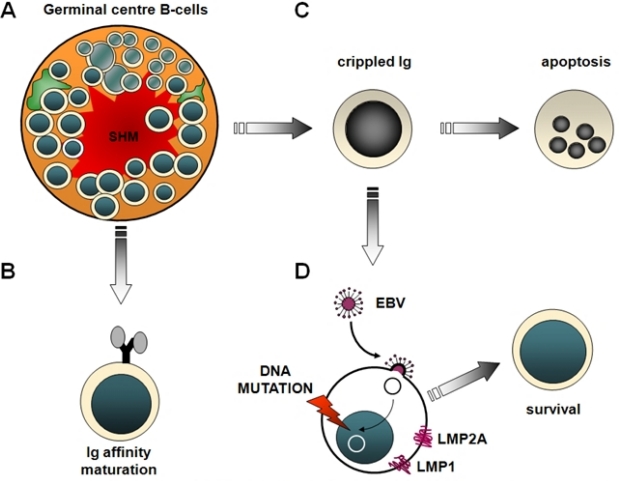
***Pathogenetic model of post-transplant lymphoproliferative disorders***. **(A)** The somatic hypermutation process (SHM) affecting the immunoglobulin variable region genes within the germinal centre is aimed at increasing the affinity of the B-cell receptor (BCR) for the antigen. **(B)** B-cells that have successfully undergone affinity maturation for the antigen are positively selected and rescued from apoptosis. **(C)** On the contrary, B-cells failing this process are induced to apoptosis. **(D)** A relevant fraction of post-transplant lymphoproliferative disorders (PTLD) fail to express the BCR, mainly due to crippling mutations of immunoglobulin genes introduced by the SHM process, and thus are predisposed to programmed cell death. The putative mechanisms that rescue PTLD from apoptosis are represented by LMP1 expression and/or molecular lesions affecting genes that regulate apoptosis.

Clues of the pathogenetic role of antigen stimulation may be derived from the molecular features of IGV genes utilized by B-cell lymphoma^93^,^94^. Among immunodeficiency-related lymphomas, HIV-related non-Hodgkin lymphomas (HIV–NHL) provide an example of antigen stimulation in disease pathogenesis^95^. The pathogenetic role of antigen stimulation in PTLD is less evident, since ∼50% PTLD derive from B-cells that have lost the ability to express a functional B-cell receptor (BCR)^20^,^21^,^96^–^98^. A frequent cause of BCR inactivation in PTLD is represented by crippling mutations of IGV genes, that are generated by the SHM process and introduce stop codons in originally in-frame rearrangements^20^,^21^,^96^–^98^. Since the expression of a functional BCR is crucial for B-cell survival, PTLD lacking BCR are thought to acquire the ability to escape apoptotic death in the absence of antigen stimulation^20^,^21^,^96^,^97^. EBV infection has been proposed as a mechanism of apoptotic rescue in PTLD with non-functional BCR, although other mechanisms might also be involved (Figure 4).

Among the 50% PTLD displaying a functional BCR, molecular signs of antigen stimulation are documented in a fraction of cases. In fact, approximately 60% PTLD with functional BCR select mutations in order to maintain intact the IGV framework region structure, and 30% select mutations to increase antigen binding affinity^20^,^98^. Overusage of specific IGV genes known to be involved in autoimmune phenomena, as observed in HIV–NHL, does not appear to be a distinctive feature of PTLD^98^. IGV mutations generated by SHM may introduce new sites of oligosaccharide linkage on the Ig protein^99^. In the immunocompetent host, this phenomenon is specific for B-NHL derived from GC cells^99^. Glycosylation may alter the biochemical properties of the Ig by enhancing or reducing the affinity for antigen. Furthermore, it might activate the BCR in an antigen independent way by mediating the interaction with lectins of the microenvironment. Despite their origin from GC B-cells, and at a variance with HIV–NHL, the acquisition of novel sites of IGV glycosylation is a rare event in PTLD^100^. This observation is consistent with the hypothesis that BCR stimulation does not play a major pathogenetic role in many PTLD.

## Molecular Histogenesis Of Ptld:

**Figure 5.**
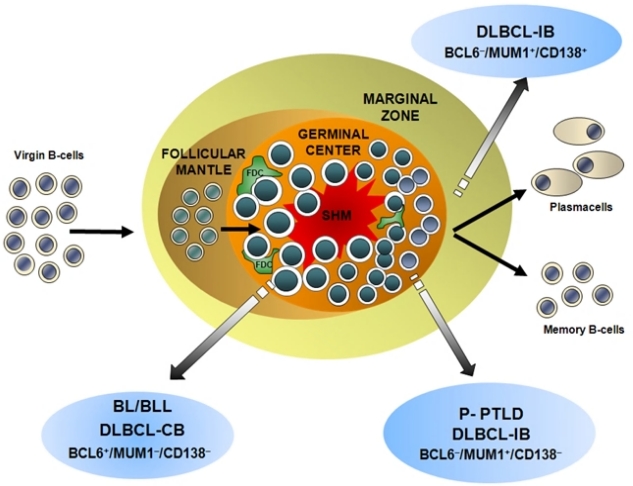
***Model of PTLD histogenesis based on the expression of the phenotypic markers BCL6, MUM1 and CD138***. The majority of PTLD, namely diffuse large B-cell lymphoma showing immunoblastic morphology (DLBCL-IB) and polymorphic PTLD (P-PTLD), carry the BCL6−/MUM1+/CD138− profile, denoting a late centrocyte origin. A fraction of DLBCL-IB are BCL6−/MUM1+/CD138+, denoting an immunoblastic-plasmablastic origin. Burkitt/Burkitt like lymphoma (BL/BLL) and diffuse large B-cell lymphoma with centroblastic morphology (DLBCL-CB) are BCL6+/MUM1−/CD138−, denoting a centroblast-centrocyte origin.

The histogenesis of PTLD has been elucidated by the application of a model exploiting genotypic and phenotypic markers and allowing the distinction of mature B-cells into different compartments, namely virgin B-cells, germinal centre (GC) B-cells and post-GC B-cells (Figure 5)^20^–^21^,^100^. The most informative genotypic marker is represented by SHM of immunoglobulin variable (IGV) genes, that takes place in the GC microenvironment^87^. Positivity for IGV SHM indicates that a given B-cell tumor derives from GC or post-GC B-cells. The presence of ongoing IGV mutations, documented by intraclonal heterogeneity, indicates that the lymphoma clone reflects centroblasts experiencing the GC reaction, whereas absence of intraclonal heterogeneity suggests derivation from late centrocytes or post-GC B-cells that have terminated the GC reaction^87^. Phenotypic markers of histogenesis include the BCL6, MUM1, and CD138 proteins, and contribute to the distinction between GC and post-GC B-cells^101^–^104^. Expression of BCL6 clusters with the GC stage of differentiation, MUM1 positivity clusters with B-cells exiting the GC and with post-GC B cells, and CD138 is a marker of pre-terminal B-cell differentiation^101^–^104^. Application of this histogenetic model to PTLD arising after solid organ transplantation has revealed that: (i) 25% P-PTLD and 10% DLBCL carry unmutated IGV genes, denoting a pre-GC origin; (ii) 100% BL and 25% DLBCL, mainly of centroblastic morphology, carry ongoing IGV mutations, denoting an origin from GC centroblasts; (iii) 75% P-PTLD and 65% DLBCL carry stable IGV mutations, denoting a centrocyte or post-GC origin^20^,^21^,^98^. The fact that IGV mutations occur in the overwhelming majority of PTLD documents that malignant transformation targets GC B-cells and their descendants both in Epstein-Barr virus (EBV)–positive and EBV–negative cases^20^,^21^,^96^–^98^. These same cellular subsets also give rise to most B-cell lymphomas in immunedeficiency settings other than post-transplant, including AIDS and primary immunodeficiencies^95^,^105^. The few PTLD lacking IGV SHM tend to arise early after transplantation, consistently carry EBV infection, mimic a post-GC phenotypic profile, and may derive from truly pre-GC B-cells or, alternatively, from B-cells that have transited through the GC but have been impaired in exerting a full GC-reaction^87^,^101^–^104^. Among PTLD deriving from GC-experienced B-cells, analysis of phenotypic markers of histogenesis identifies three predominant profiles of the disease (Figures 1 and 5)^21^. PTLD belonging to the first histogenetic category express the BCL6+/ MUM1+/-/CD138− profile and reflect B-cells actively experiencing the GC reaction. These PTLD associate with ongoing SHM and are morphologically classified as DLBCL centroblastic or as BL^21^. A second category of PTLD reflects the BCL6−/MUM1+/CD138-phenotype and comprises 65% P-PTLD and 30% DLBCL, mainly with immunoblastic features^21^. This PTLD subset putatively derives from B-cells that have concluded the GC reaction but have not yet undergone terminal differentiation. The BCL6−/MUM1+/CD138− profile is common among PTLD, but is rare among HIV-related lymphomas, underscoring biological differences between these two groups of immunodeficiency-related lymphomas^21^,^101^. A third group of PTLD is reminiscent of post-GC and pre-terminally differentiated B-cells and show the BCL6−/MUM1+/CD138+ phenotype and, if EBV positive, express the LMP1 antigen 21. These PTLD are morphologically represented by either P-PTLD (35% of cases) or DLBCL immunoblastic. The BCL6−/MUM1+/CD138+ histogenetic profile is shared also by many HIV-related lymphomas^101^.
